# Improved Glucagon Sensitivity Following Weight Loss: A Systematic Review and Meta‐Analysis

**DOI:** 10.1111/obr.70167

**Published:** 2026-05-18

**Authors:** Hye‐Rin Charlotte Kim, Lima Hamidi, Anne Majumdar, Matyas Fehervari, Emma Rose McGlone

**Affiliations:** ^1^ Department of Metabolism, Digestion and Reproduction Imperial College London London UK; ^2^ London North West University Healthcare NHS Trust London UK; ^3^ Department of Health Studies Royal Holloway University of London Egham UK; ^4^ Department of Surgery and Cancer Imperial College London London UK; ^5^ Department of Bariatric Surgery Maidstone and Tunbridge Wells NHS Trust Tunbridge Wells UK; ^6^ Department of Bariatric Surgery Whittington Hospital NHS Trust London UK

**Keywords:** bariatric surgery, glucagon, obesity, weight loss

## Abstract

**Objective:**

This systematic review and meta‐analysis investigated the effect of weight loss in people with obesity on two biomarkers of glucagon resistance: fasting plasma glucagon and alanine.

**Methods:**

A comprehensive search was conducted in Medline and Embase. Random‐effects meta‐analyses and correlation analyses were performed. Risk of bias was assessed using the Cochrane RoB 2 and MINORS tools.

**Results:**

Forty‐seven studies comprising 69 interventions and 2061 participants were included. Pooled mean weight loss was −18.23 kg (95% CI [−20.20, −16.27]). Both fasting glucagon and alanine decreased following weight loss: −4.05 pmol/L (95% CI [−4.50, −3.60]; *p* < 0.001) and −17.9% (95% CI [−22.7, −13.1]; *p* < 0.0001), respectively. Glucagon reduction occurred with all weight‐loss intervention types: surgical −3.85 pmol/L (95% CI [−5.48, −2.22]; *p* < 0.0001), dietary −2.87 pmol/L (95% CI [−4.20, −1.54]; *p* < 0.0001), and pharmacological −5.32 pmol/L (95% CI [−9.85, −0.78]; *p* < 0.0001). Greater weight loss correlated with larger reductions in fasting glucagon (Pearson *r* = 0.3255, *p* = 0.0064). Heterogeneity was high, and overall risk of bias was moderate.

**Conclusions:**

Weight loss is associated with significant reductions in fasting glucagon and alanine concentrations, suggesting improved hepatic glucagon sensitivity and partial restoration of the liver–α‐cell axis.

**Abbreviations**
ELISA: enzyme‐linked immunosorbent assay
GIP: glucose‐dependent insulinotropic polypeptide
GLP‐1/GLP‐2: glucagon‐like peptide‐1/‐2
GLP‐1RA: glucagon‐like peptide‐1 receptor agonist
MASLD: metabolic dysfunction–associated steatotic liver disease
RIA: radioimmunoassay
RYGB: Roux‐en‐Y gastric bypass
SG: sleeve gastrectomy
SGLT2: sodium‐glucose cotransporter‐2
T2D: type 2 diabetes

## 1 Introduction

Metabolic dysfunction–associated steatotic liver disease (MASLD) now affects more than one in three adults globally [1]. It is closely associated with other metabolic conditions in prevalence—75% of people with obesity have MASLD and 69% of people with T2D have MASLD—and pathogenesis [2]. While considerable attention has historically been given to the role of hepatic and peripheral insulin resistance in the development of MASLD, obesity, and T2D [3], aberrant glucagon signaling is gaining recognition as another contributor to the metabolic dysfunction underpinning these conditions [4].

Glucagon is an important regulator of glucose homeostasis, lipid and amino acid metabolism, and energy regulation [5]. Under physiological conditions, glucagon stimulates hepatic amino acid uptake and catabolism via the urea cycle, facilitating the clearance of circulating amino acids and the production of intermediaries for gluconeogenesis [6]. The secretion of glucagon from pancreatic α‐cells is responsive to nutritional status; one powerful stimulus to glucagon secretion is circulating amino acids [7]. Thus, a feedback relationship exists between plasma amino acids and glucagon, known as the liver–α‐cell axis (Figure 1A) [8, 9, 10, 11].

**FIGURE 1:**
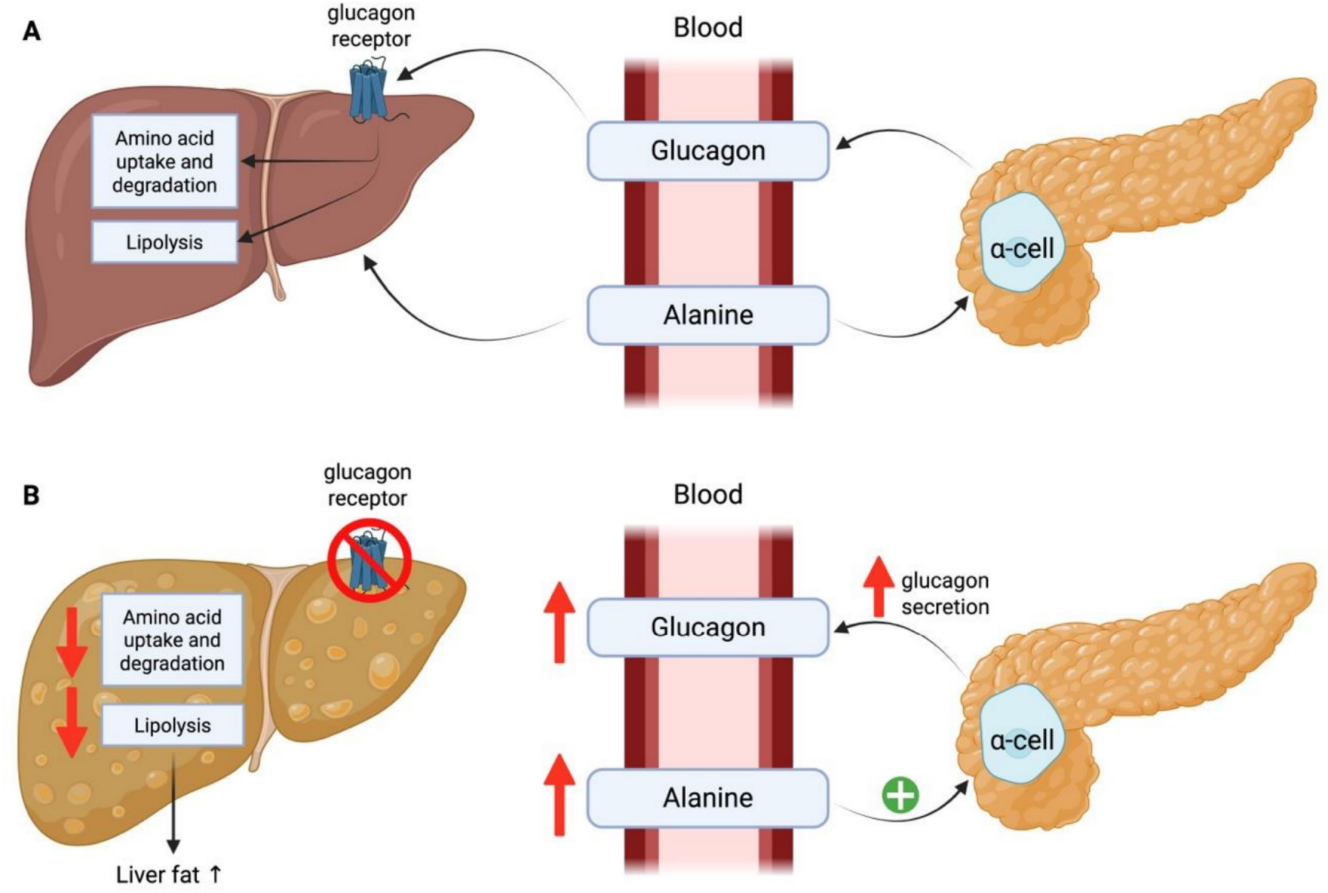
Schematic overview of the liver–α‐cell axis and its disruption in glucagon resistance associated with obesity and MASLD. (A) Under physiological conditions, circulating alanine and other amino acids stimulate glucagon secretion from pancreatic α‐cells. Glucagon binds to hepatic glucagon receptors, promoting amino acid uptake and degradation via ureagenesis. Hepatic glucagon signaling also decreases lipid accumulation. (B) In obesity and MASLD, impaired hepatic glucagon receptor signaling reduces amino acid metabolism, leading to elevated circulating alanine and other amino acids in the blood. Persistent α‐cell stimulation results in hyperglucagonemia and hyperaminoacidemia, while decreased glucagon‐mediated lipolysis contributes to hepatic fat accumulation. This figure was created using BioRender (Kim, H. (2025) https://BioRender.com/nve2qx0).

In people with MASLD and/or obesity, this feedback loop becomes disrupted [4, 12, 13]. Hepatocytes are less responsive to glucagon, resulting in impaired hepatic amino acid uptake and disposal and leading to inappropriately elevated fasting levels of amino acids (Figure 1B) [14, 15, 16]. The consequent hyperaminoacidemia stimulates the pancreas, driving α‐cell hyperplasia and excessive glucagon secretion, reinforcing a maladaptive cycle of hyperglucagonemia and hyperaminoacidemia [11, 17, 18]. Gluconeogenic amino acids such as alanine are particularly affected by this phenomenon [4]. Because glucagon also acts on the liver to decrease de novo lipogenesis and stimulate lipolysis, resistance to glucagon is likely to exacerbate hepatic fat accumulation [19].

Consequently, fasting plasma glucagon and fasting plasma alanine have been identified as biomarkers of glucagon resistance [20]. These markers are consistently elevated in individuals with obesity, MASLD, and other forms of metabolic disease [12, 16, 21, 22]. Although it is well evidenced that robust and sustained weight loss leads to improvement or resolution of MASLD and T2D [2], the effect of body weight change on glucagon resistance remains unclear.

This systematic review and meta‐analysis aimed to evaluate whether clinically significant weight loss in individuals with obesity results in significant improvements in biomarkers of glucagon resistance, specifically fasting glucagon and fasting alanine levels. Furthermore, we hypothesized that there is a dose‐dependent relationship between weight loss and biomarkers of glucagon resistance, with no difference observed between surgical, dietary, and pharmacological interventions for weight loss.

## 2 Methods

This systematic review and meta‐analysis was conducted following the Preferred Reporting in Systematic Reviews and Meta‐Analyses (PRISMA) [23] guidelines 2020 and adhered to the Cochrane Handbook for Systematic Reviews of Interventions [24]. The review protocol was registered in PROSPERO (https://www.crd.york.ac.uk/PROSPERO/) with the registration number CRD42024599458.

### 2.1 Search Strategy and Selection Criteria

Studies were identified through a comprehensive electronic search of MEDLINE and Embase databases including the following terms: glucagon; alanine; fasting; weight loss; dieting; bariatric surgery; anti‐obesity agents; glucagon‐like peptide‐1 receptor agonists. The final search was conducted on November 18, 2024. All results from both databases were imported into Covidence [25], a web‐based collaboration software platform that streamlines the production of systematic and other literature reviews. We used it for screening and data extraction.

Two reviewers (H.C.K. and L.H.) independently assessed studies for inclusion via title, abstract, and full text using the online Covidence tool. Discrepancies were resolved by discussion and input from a third reviewer (E.R.M.).

Studies were included if they were interventional or cohort designs (single or multiple arm). Studies in languages other than English were excluded. Study subjects were adults and children aged 12 years or over, male and female with overweight or obesity (BMI > 25 kg/m^2^). Cohorts with type 1 diabetes and end‐stage renal disease, decompensated or active viral liver disease, previous nonbariatric major gut resection, and active cancer were excluded due to potential derangement of gut hormones and cachexia of tumor. Eligible studies reported on dietary, pharmacological, endoscopic or surgical methods of intentional weight loss and each intervention achieved a minimum of mean 5% total body weight loss over a duration of at least 4 weeks. For glucagon measurement, we included only studies using either ELISAs that reported no cross‐reactivity with oxyntomodulin, glicentin, GLP‐1, and GLP‐2, or radioimmunoassays (RIA) targeting the C‐terminus with validated sensitivity [26].

### 2.2 Data Collection

Data collection was carried out by H.C.K. according to the prespecified criteria and was reviewed and verified by all authors at different stages to ensure accuracy and completeness of the collected data. Primary outcomes were defined as changes in body weight, fasting plasma glucagon and fasting plasma alanine levels. Secondary outcomes were the methods of weight reduction, including surgical, dietary, and pharmacological interventions.

Data relevant to these were systematically extracted using Covidence software and a standardized Excel template created for this review. Extracted information included the article title, authors, year of publication, country of origin, study design, sample size, duration of the intervention, and the primary outcomes (body weight, fasting plasma glucagon and alanine, at both baseline and endpoint). For the secondary outcomes, details on the interventions were recorded, which included specifying the surgical method in case of bariatric surgery, and dosage and frequency for pharmacological weight‐loss agents. Additionally, glucagon assay types were recorded. Primary outcomes were extracted as the absolute change in body weight in kilograms from baseline to endpoint, as well as changes in fasting plasma glucagon and alanine levels. If standard deviations were not reported, interquartile range or SE were used to estimate standard deviation, and units were standardized.

### 2.3 Risk of Bias Assessment

Risk of bias was independently assessed by two reviewers (H.C.K. and L.H.). The risk of bias for randomized controlled trials was evaluated using the Cochrane Risk of Bias 2 (RoB 2) tool [27], while nonrandomized interventional studies were assessed using the Methodological Index for Non‐Randomized Studies (MINORS) [28]. The MINORS tool was selected because it was specifically developed for nonrandomized surgical and interventional studies, including both single‐arm and comparative designs. Given that blinding is often not feasible in surgical studies, the MINORS criteria account for this limitation [28].

### 2.4 Data Analysis

All analyses were performed using STATA version 18.5 (StataCorp. 2025. Stata Statistical Software: Release 18.5. College Station, TX: StataCorp LLC). Meta‐analyses were performed to assess changes in body weight, fasting plasma glucagon, and alanine. To account for differences in study design and risk of bias, two complementary analyses were performed as follows: (1) RCTs with a concurrent control group (placebo or standard care) were analyzed by pooling between‐group differences in change from baseline, and (2) all study arms from both randomized and nonrandomized interventional studies were analyzed by pooling within‐group changes from baseline across all intervention arms to estimate the overall effect of intervention exposure. Effect sizes were pooled using weighted mean differences (WMD) with 95% confidence intervals. Results were considered as significant when *p* < 0.05. DerSimonian and Laird random‐effects model was used for meta‐analyses to account for heterogeneity across studies. Meta‐analyses were visualized using Forest plots. Heterogeneity was quantified using *I* ^2^ statistic. Subgroup analyses by intervention type were performed to address heterogeneity of the changes in body weight and biomarkers of glucagon sensitivity. Pearson and Spearman correlation analyses were conducted to examine the association between weight loss and reductions in fasting plasma glucagon and alanine. Random‐effects meta‐regression was performed to assess whether the magnitude of change in body weight was associated with corresponding changes in biomarkers of glucagon resistance. Unlike simple linear regression, meta‐regression assigns greater weight to studies that provide reliable effect estimates, usually larger studies. Moreover, the random‐effects model allows for residual heterogeneity, which provides more reliable estimates [29]. Stratified meta‐regression analyses by intervention type were also performed to identify any patterns in dose‐dependency.

## 3 Results

### 3.1 Study Selection

A total of 3869 studies were identified through the initial search, as shown in Figure 2, including 2295 from MEDLINE and 1574 from Embase. After removal of duplicates, 2606 abstract were screened and of these, 417 studies were retrieved for full‐text assessment. Consequently, 369 studies were excluded for reasons such as wrong outcomes, wrong study designs, unvalidated glucagon assay, and for not meeting the predefined inclusion criteria. Therefore, 47 studies were included in the final analysis for fasting plasma glucagon, and 10 studies for the systematic review of alanine. In total, 69 individual intervention arms with 2061 participants were analyzed for glucagon, comprising 41 surgical, 14 pharmacological, and 14 dietary interventions. For the review of alanine, 14 individual interventions with 596 participants were included, comprising 2 surgical, 6 pharmacological and 6 dietary interventions (Figure 2).

**FIGURE 2:**
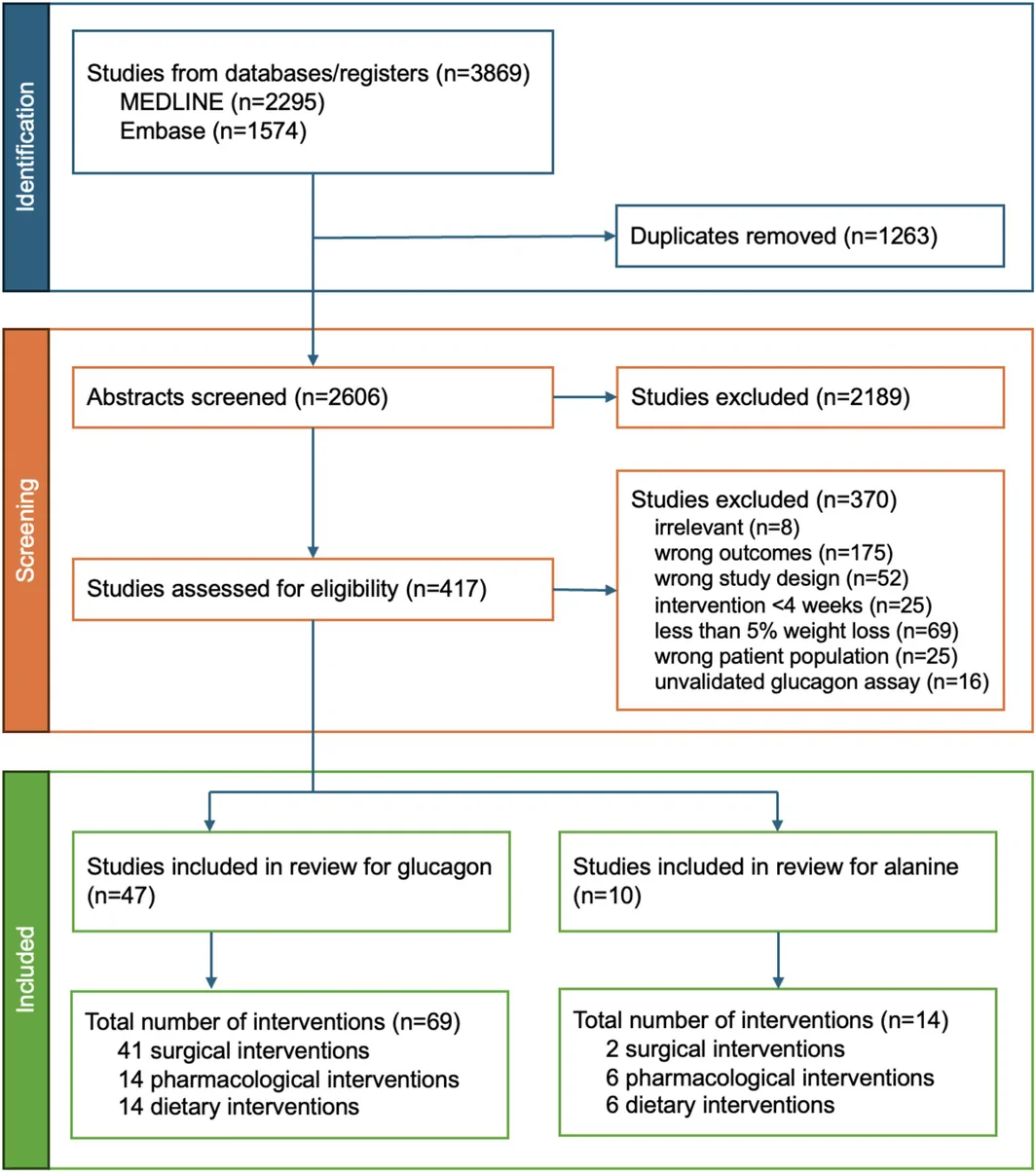
Flow diagram of study selection.

### 3.2 Study Characteristics

Table S1 provides a summary of all studies included in this review. Five studies reported exclusively on fasting alanine concentrations [30, 31, 32, 33, 34], another five reported both glucagon and alanine concentrations [33, 35, 36, 37, 38]. The included studies were published between 1984 and 2024 [32, 39]. Sample sizes ranged from 6 to 211 participants [37, 40]. The age of participants ranged from 25 to 69 years [32, 41].

Mean baseline body weight ranged from 72.2 to 163 kg [38, 40]. In studies investigating surgical weight loss, baseline body weight ranged from 100 to 163 kg [40, 42], whereas in dietary interventions, it ranged from 84 to 125 kg [32, 43]. Pharmacological interventions had a baseline range from 72.2 to 104.2 kg [37, 44]. The mean percentage of total weight loss achieved ranged from 5.1% to 35.3% [37, 45]. Intervention duration ranged from 28 days to 2 years [46, 47, 48]. Nineteen studies had a follow‐up period of 12 months or longer, almost all involving bariatric surgery interventions, with the exception of two pharmacological interventions [44, 49], and one dietary trial [48].

Roux‐en‐Y gastric bypass surgery was the most frequently performed surgical procedure, reported in 24 trials [34, 36, 40, 42, 45, 50, 51, 52, 53, 54, 55, 56, 57, 58, 59, 60, 61, 62, 63, 64, 65, 66, 67], followed by sleeve gastrectomy in seven studies [45, 56, 60, 62, 68]. Less common surgical methods included the gastric curvature plication [45], vertical banded gastroplasty [69, 70], and jejunal–ileal bypass [70]. Three studies reported on duodenal–jejunal bypass liner and these endoscopic treatments have been included in the surgical group for subgroup analysis [71, 72, 73]. Among pharmacological interventions, six studies investigated GLP‐1 receptor agonists (liraglutide [44, 74, 75], exenatide [39, 49], semaglutide) [76], two examined SGLT2 inhibitors (canagliflozin [74], dapagliflozin) [39], two assessed dual GIP/GLP‐1 receptor agonists (tirzepatide) [37, 76], and one investigated a glucagon receptor/GLP‐1 receptor dual agonist (survodutide, BI 456906) [38]. Dietary interventions were predominantly based on calorie restriction [30, 31, 41, 43, 46, 61, 77, 78, 79]. Low‐calorie diets were defined as an energy intake of approximately 800–1200 kcal/day, while very low‐calorie diets were defined as providing < 800 kcal/day. Three studies investigated paleolithic diets [35, 48, 80], and two studies compared conventional diabetes and carbohydrate‐reduced, high‐protein diets (Kjeldsen et al. [33] included two studies, which were analyzed separately).

Most studies measured fasting plasma glucagon concentrations using commercially available RIA [35, 39, 40, 41, 43, 45, 46, 47, 48, 49, 53, 55, 56, 57, 58, 59, 61, 62, 63, 65, 67, 69, 70, 72, 74, 75, 77, 78, 79], or ELISA kits [33, 36, 37, 38, 42, 44, 50, 51, 60, 64, 66, 68]. Two studies used human diabetes bio‐plex assays [71, 80], and one used a chemiluminescent sandwich immunoassay [71, 76, 80]. Alanine concentrations were measured with high‐performance liquid chromatography [30], nuclear magnetic resonance spectroscopy [31, 34], continuous primed tracer isotope infusion [32], an l‐amino acid assay kit (Abcam, ab65347) [33], and liquid chromatography‐mass spectrometry [35, 37].

### 3.3 Weight Loss is Associated With Reduction in Fasting Plasma Glucagon Concentrations

Pooled data from all intervention arms showed a significant overall reduction in total body weight with a WMD of −18.23 kg (95% CI: [−20.20, −16.27]; *p* < 0.0001; Figure S2A). Heterogeneity was high (*I* ^2^ = 99.4%), likely due to variability in intervention types and follow‐up durations. Meta‐analysis of RCTs showed an overall effect of −10.03 kg (95% CI: [−13.48, −6.57]; *p* < 0.001; Figure S2B). Subgroup analysis revealed that most weight loss was achieved following surgical interventions (−25.96 kg, 95% CI: [−28.33, −23.60]), followed by dietary interventions (−10.19 kg, 95% CI: [−12.61, −7.80]), and pharmacological therapies (−6.54 kg, 95% CI: [−7.83, −5.25]; Figure 3).

**FIGURE 3:**
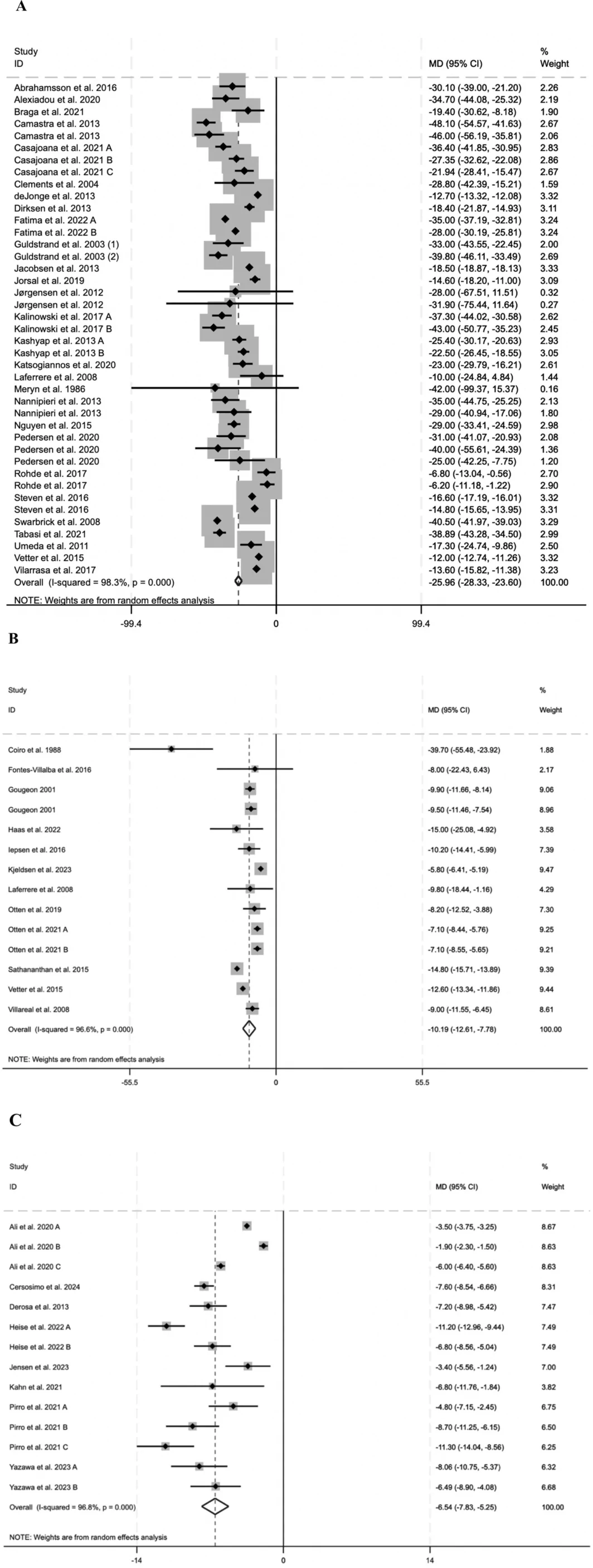
Forest plots of meta‐analyses showing effect sizes (ES) with 95% confidence intervals (CI) for changes in absolute body weight (kg) by intervention type: (A) surgical, (B) dietary, and (C) pharmacological interventions. Weights are from random‐effects analyses using the DerSimonian–Laird (DL) method.

Alongside these significant reductions in body weight, fasting plasma glucagon concentrations also decreased. Pooled data from all intervention arms found a reduction of fasting plasma glucagon with a pooled WMD of −4.05 pmol/L (95% CI [−4.50, −3.60]; *p* < 0.001; Figure S3A). Meta‐analysis of RCTs demonstrated similar effect size: −4.45 pmol/L (95% CI [−6.83, −2.07]; *p* < 0.001; Figure S3B). High heterogeneity was observed across studies (*I* ^2^ = 97.6%).

### 3.4 Reduction in Fasting Plasma Glucagon by Intervention Type

When stratified by the type of weight loss intervention, all subgroups showed significant reduction in fasting glucagon levels. Surgical procedures had a mean reduction of −3.85 pmol/L (95% CI: [−5.48, −2.22]; *p* < 0.0001; Figure 4A), dietary interventions resulted in a reduction of −2.87 pmol/L (95% CI: [−4.20, −1.54]; *p* < 0.0001; Figure 4B), and pharmacological treatments showed the greatest change (−5.32 pmol/L; 95% CI: [−9.85, −0.78]; *p* < 0.0001; Figure 4C).

**FIGURE 4:**
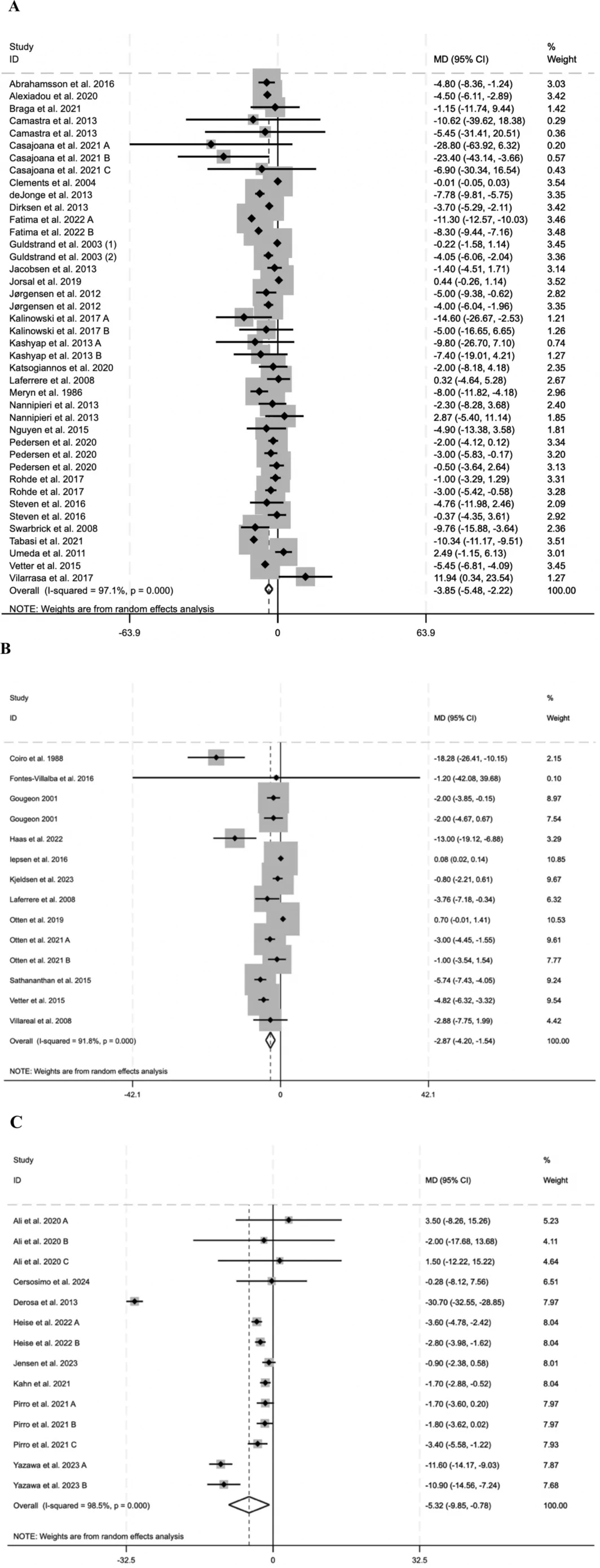
Forest plots of meta‐analyses showing effect sizes (ES) with 95% confidence intervals (CI) for changes in fasting plasma glucagon (pmol/L) by intervention type: (A) surgical, (B) dietary, and (C) pharmacological interventions. Weights are from random‐effects analyses using the DerSimonian–Laird (DL) method. Heterogeneity was measured by *I* ^2^. Weight corresponds to the percentage of contribution of each intervention to the overall effect.

### 3.5 Greater Weight Loss is Associated With Greater Reductions in Fasting Plasma Glucagon

A moderate‐sized positive correlation between weight loss and reduction in fasting plasma glucagon was observed, with Pearson's correlation coefficient of *r* = 0.3255 (*p* = 0.0064; Figure 5). Spearman's rank correlation demonstrated a similar positive monotonic association (rho = 0.4328, *p* = 0.0002).

**FIGURE 5:**
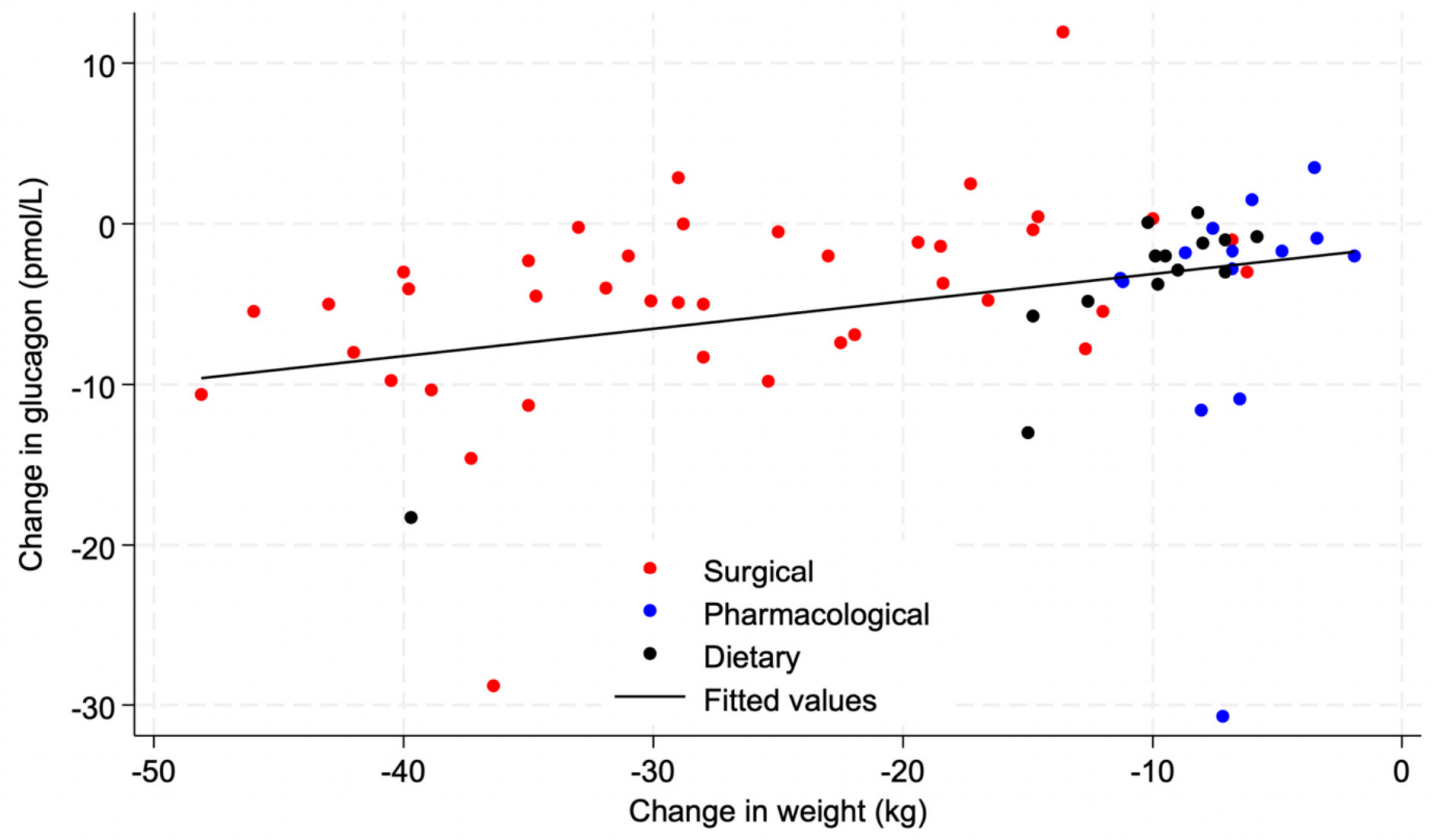
Scatter plot showing the Pearson correlation between weight change (kg) and changes in fasting glucagon (pmol/L). Each dot represents an individual study arm, color‐coded by intervention type: surgical (red), pharmacological (blue), dietary (green). The black line indicates the fitted regression line. A significant positive correlation was observed (*r* = 0.33, *p* = 0.006).

To further investigate whether there was a dose‐dependent relationship between the extent of weight loss and the reduction in fasting plasma glucagon, we performed meta‐regression of changes in body weight on improvements in glucagon. Meta‐regression with all intervention arms pooled revealed a positive association between weight loss and glucagon reduction (95% CI [−0.06, 0.73]), although this did not achieve statistical significance (*p* = 0.093).

When stratified by intervention type, the strongest correlation was observed for dietary interventions (*r* = 0.8884, *p* < 0.0001). Surgical interventions showed a moderate correlation (*r* = 0.4023, *p* = 0.0091), whereas pharmacological interventions showed no correlation (*r* = 0.2044, *p* = 0.4834).

Stratified meta‐regression by intervention type indicated a dose‐dependency in both dietary and surgical interventions. In the case of dietary weight loss, each kilogram of weight loss was associated with an average reduction in fasting plasma glucagon of 0.56 pmol/L (*p* < 0.001). Surgical interventions also showed a significant association, with each kilogram of weight loss correlating with a reduction of 0.17 pmol/L in fasting glucagon (*p* = 0.005). Again, no significant association was observed for pharmacological interventions (*β* = 2.16, *p* = 0.488).

### 3.6 Weight Loss is Associated With Reduction in Fasting Plasma Alanine

Ten studies comprising 596 participants reported changes in fasting plasma alanine following weight loss. In total, 14 individual study arms reported changes in fasting plasma alanine after weight loss, and 13 reported on total body weight loss (while Lopes et al. [34] did not provide absolute weight data, it reported clinically relevant improvements in BMI and was therefore included in the alanine analysis). Meta‐analysis showed a body weight reduction of −7.45 kg (95% CI [−9.62, −5.29]; *p* < 0.0001; Figure S4A), alongside a mean percentage decrease in plasma alanine of −17.92% (95% CI [−22.71, −13.13]; *p* < 0.0001; Figure S4B).

### 3.7 Risk of Bias

Among the 20 randomized controlled trials, the overall risk of bias was low to moderate across most studies (Figure S1). Eight studies were judged to have a low risk of bias, ten studies had a moderate risk, and two studies had a high risk, mostly due to concerns in Domain 5 (bias in selection of the reported results). This was attributed to the absence of trial registration information. For nonrandomized studies, total MINORS scores ranged from 10 to 13 out of a maximum of 16 for single‐arm studies and from 17 to 18 out of 24 for comparative studies (Table S2). Moderate quality was most commonly due to the lack of prospective calculation of study size and incomplete inclusion of consecutive patients.

## 4 Discussion

This systematic review and meta‐analysis examined the relationship between weight loss and biomarkers of glucagon resistance in individuals with overweight or obesity. Fasting glucagon and alanine are elevated in people with MASLD and obesity [4, 13, 15, 17, 81], which is thought to be due to an aberrant liver–alpha cell axis: (1) hepatocytes do not respond effectively to glucagon, (2) alanine is not cleared and metabolized from the blood, and (3) alanine persistently stimulates alpha cells to secrete glucagon [4, 11]. Our analysis found that weight loss was associated with reductions in both biomarkers, suggesting that losing weight is associated with improved glucagon sensitivity. Moreover, we found that fasting glucagon decreases irrespective of the method of weight loss; and that there was a correlation between the amount of weight loss and the reduction in plasma glucagon for both surgical and dietary weight loss. These findings suggest that glucagon resistance is reversible and that interventions leading to clinically meaningful weight loss can improve glucagon signaling in people with obesity.

The effect size of glucagon reduction (4 pmol/L) is likely to be clinically meaningful, given that fasting levels of glucagon are around 6–7 pmol/L in the lean population and around 12 pmol/L in those with MASLD [16, 82]. This reduction in fasting glucagon following weight loss corroborates the findings of a 2017 systematic review and meta‐analysis [83]. We found that since this previous review, the number of studies recording weight loss alongside plasma glucagon levels has more than doubled. The present analysis included studies in which weight loss was achieved by pharmacological means, a cohort not included in the previous meta‐analysis. Furthermore, although a correlation between amount of weight loss and reduction in plasma glucagon was previously reported, in the previous analysis most of the dietary interventions resulted in less than 3% weight loss, limiting the generalizability of this finding. In the present analysis, we chose only to include studies reporting at least 5% weight loss in each intervention group analyzed. We also analyzed changes in plasma alanine and noted a significant reduction following weight loss. This suggests improved hepatic amino acid uptake and degradation. The synergistic decrease in both alanine and glucagon concentrations strongly supports the notion that weight loss is associated with a restored liver–α‐cell feedback cycle.

The mechanism by which hepatic resistance to glucagon occurs is not yet clear. It may result from reduced glucagon receptor expression at the cell membrane, alterations in signaling properties of glucagon receptor, or reduced downstream signaling through pathways regulating amino acid metabolism and lipid oxidation [4]. Such changes could arise secondary to hepatic steatosis and metabolic stress, contributing to impaired glucagon responsiveness in MASLD. Although we did not directly investigate liver fat in the participants included in this review, weight loss is strongly associated with a reduction in liver fat, and this relationship is dose‐dependent [84, 85]. Our observation that weight loss is associated with significant improvement in markers of glucagon resistance could indicate that reduction in hepatic steatosis improves signaling at the level of the glucagon receptor. Previous research has demonstrated that hepatic cholesterol content is negatively correlated with glucagon sensitivity [86]. One possible mechanistic explanation for our findings is that a reduction in hepatic steatosis alters the lipid content of the cell membrane, which changes glucagon receptor conformation and signaling properties, either directly via an allosteric effect or indirectly via modulating biophysical properties of the membrane [87]. Alternatively, an improvement in glucagon sensitivity could also cause a reduction in liver fat, given that glucagon strongly stimulates hepatic fat clearance, via a reduction in de novo lipogenesis and increased lipolysis [20, 88]. In reality, the observed changes in markers of glucagon sensitivity may be both the cause and effect of weight loss, with improved liver health restoring a functional negative feedback cycle.

Interestingly, although we saw a strong correlation between weight loss and reduction in plasma glucagon for weight loss achieved via both surgery and diet, this relationship was not observed for pharmacological weight loss. Although pharmacological treatment was associated with the least weight loss, the reduction in plasma glucagon was greater than for surgical and dietary interventions. One explanation for this could be that most of the medications included in this review were GLP‐1RAs, and one of the effects of GLP‐1R agonism is to suppress glucagon secretion from alpha cells during hypoglycemia [89]. Thus, for GLP‐1RA‐induced weight loss, reduced fasting plasma glucagon may reflect both an improvement in glucagon sensitivity and a direct suppression of glucagon release.

We acknowledge several limitations to this review and meta‐analysis. The included studies varied substantially in design, duration, and population characteristics, resulting in high heterogeneity. Although there is substantial overlap in prevalence of MASLD and obesity, it is likely that MASLD drives glucagon resistance [4, 19]. Thus this analysis might have found less heterogenous results had it been designed to correlate reduction in liver fat with biomarkers of glucagon resistance. This approach, however, would have dramatically reduced the numbers of studies available for inclusion. Furthermore, in this study, we chose to focus on plasma alanine as a representative gluconeogenic amino acid that has been relatively well studied. Other relevant amino acids that could also have been studied would include arginine, proline and glycine [14, 90]. Crucially, we acknowledge that fasting glucagon and alanine are surrogate markers of glucagon resistance. While these markers are associated with hepatic glucagon action, they do not provide direct mechanistic evidence of a change in glucagon signaling. Future studies should assess whether reductions in liver fat and/or body weight are associated with improvements in glucagon resistance, as directly measured, for example, in mechanistic clamp studies.

## 5 Conclusion

This systematic review and meta‐analysis finds that clinically significant weight loss in people with obesity is associated with improvements in biomarkers of glucagon resistance. Bariatric surgery, diet, and pharmacological agents all caused significant weight loss that was associated with a decrease in fasting plasma glucagon and alanine. These findings suggest that hepatic glucagon sensitivity can be at least partially restored through body weight reduction. The dose‐dependent relationship observed in surgical and dietary interventions further supports the importance of achieving and maintaining clinically meaningful weight reduction to improve metabolic outcomes in individuals with obesity and MASLD.

## Funding

E.R.M. is supported by the NIHR.

## Conflicts of Interest

The authors declare no conflicts of interest.

## Supporting information


**Table S1:** Summary of studies where glucagon and/or alanine were measured alongside body weight. Concentrations of glucagon and alanine have been converted where necessary to be presented in pmol/L. The change of alanine is shown as a percentage (%). All results are presented as mean ± SD. B, baseline; *Δ*, change from baseline to endpoint (%); PCS‐S, single‐arm prospective cohort study; PCS‐C, controlled prospective cohort study; (m)RYGB, modified Roux‐en‐Y gastric bypass; (L)SG, (laparoscopic) sleeve gastrectomy; GCP, gastric curvature plication; DJBL, duodenal–jejunal bypass liner; CD, conventional diabetes diet; CRHP, carbohydrate‐reduced high‐protein diet; (V)LCD, (very) low‐calorie diet***; MET, metformin; ELISA, enzyme‐linked immunosorbent assay; RIA, radioimmunoassay; LC–MS(/MS), liquid chromatography(–tandem) mass spectrometry. *, Studies reporting on alanine; **, Studies reporting on both alanine and glucagon; ***, LCD defined as 800–1200 kcal/day; VLCD defined as < 800 kcal/day.
**Table S2:** Risk of bias assessment of included nonrandomized studies using MINORS.
**Figure S1:** Risk of bias assessment of included RCTs using the Cochrane RoB 2 tool and proportion of studies judged as low risk, some concerns, or high risk of bias across each domain and for overall risk of bias. Visualized with robvis tool.
**Figure S2:** (A) Forest plot presenting meta‐analysis of effect sizes (ES) with 95% confidence intervals (CI) for changes in absolute body weight (kg) across all intervention arms (*n* = 69). The pooled overall effect estimate was derived using DerSimonian–Laird (DL) method. Heterogeneity was measured by I^2^. Weight corresponds to the percentage of contribution of each intervention to the overall effect. (B) Forest plot presenting random‐effects meta‐analysis of RCTs, using the restricted maximum likelihood (REML) method. The plot presents the mean difference and standard deviation (SD) in absolute body weight (kg) between treatment and control groups, with 95% confidence intervals (CI). Each square represents an individual comparison, with the size proportional to its weight in the analysis, and horizontal lines denote 95% CI. The diamond indicates the pooled overall effect (mean difference −10.03 kg; 95% CI [−13.48, −6.57]; p < 0.001). Between‐study heterogeneity was substantial (τ2 = 44.29, I^2^ = 95.1%, p < 0.001).
**Figure S3:** (A) Forest plot presenting meta‐analysis of effect sizes (ES) with 95% confidence intervals (CI) for changes in fasting plasma glucagon (pmol/L) across all intervention arms (*n* = 69). The pooled overall effect estimate was derived using DerSimonian–Laird (DL) method. Heterogeneity was measured by I^2^. Weight corresponds to the percentage of contribution of each intervention to the overall effect. (B) Forest plot presenting random‐effects meta‐analysis of RCTs, using the restricted maximum likelihood (REML) approach. The plot presents the mean difference and standard deviation (SD) in fasting plasma glucagon (pmol/L) between treatment and control groups, with 95% confidence intervals (CI). Each square represents an individual comparison, with the size proportional to its weight (%) in the analysis, and horizontal lines denote 95% CI. The diamond indicates the pooled overall effect (mean difference −4.45 pmol/L; 95% CI [− 6.83, −2.07]; p < 0.001). Between‐study heterogeneity was substantial (τ2 = 44.29, I^2^ = 95.1%, p < 0.001).
**Figure S4:** Forest plots presenting meta‐analyses of the mean difference in (A) absolute body weight (kg) and (B) fasting alanine levels (% change from baseline) following weight loss interventions. Dietary interventions are shown in green, pharmacological interventions in blue and surgical interventions in red. Heterogeneity was measured by I^2^. Weight corresponds to the percentage of contribution of each intervention to the overall effect.

## Data Availability

The data that support the findings of this study are available from the corresponding author upon reasonable request.
