# Supplementary material for: Improved Glucagon Sensitivity Following Weight Loss: A Systematic Review and Meta‐Analysis
Source: Obes Rev. 2026 May 18;27(12):e70167. doi: 10.1111/obr.70167 (PMC13749330; doi:10.1111/obr.70167)
Supplement: Supplementary file 1 [file OBR-27-e70167-s001.docx]

*Supplementary Information*

**Improved Glucagon Sensitivity following Weight Loss:**

**A Systematic Review and Meta-Analysis**

**Authors:**

Hye-Rin Charlotte Kim^1^, Lima Hamidi^2^, Anne Majumdar^1,3^, Matyas Fehervari^4,5^, Emma Rose McGlone^4,6*^

**Affiliations:**

^1^Department of Metabolism, Digestion and Reproduction, Imperial College London, London, UK

^2^London North West University Healthcare NHS Trust, London, UK

^3^Dept of Health Studies, Royal Holloway University of London, Egham, UK

^4^Department of Surgery and Cancer, Imperial College London, London, UK

^5^Department of Bariatric Surgery, Maidstone and Tunbridge Wells NHS Trust, Tunbridge Wells, UK

^6^Department of Bariatric Surgery, Whittington Hospital NHS Trust, London, UK

***Corresponding author:** Emma Rose McGlone, [e.mcglone@imperial.ac.uk](mailto:e.mcglone@imperial.ac.uk)

**Table S1. Summary of studies included in the meta-analysis**

| Study ID | Country | Design | Participants | Intervention | Duration | BWT (kg) | Glucagon (pmol/L) | Alanine (%Δ) | Assay Type |
| --- | --- | --- | --- | --- | --- | --- | --- | --- | --- |
| Abrahamsson et al. 2016 | Sweden | PCS-S | n = 12; Age = 43.1 ± 10.8 years; 8 F and 4 M | RYGB | 4 - 5 months | B = 116.5 ± 15.7; 4M = 86.4 ± 16.2; Δ = 25.83% | B = 13.4 ± 6.9; 4M = 8.6 ± 5.4 | NA | ELISA |
| Alexiadou et al. 2020 | UK | PCS-S | n = 19; Age = 49.7 ± 13.0 years; 16 F and 3 M | RYGB | 12 months | B = 115.3 ± 25.3; 12M = 80.6 ± 16.3; Δ = -30.1% | B = 10.7 ± 3.74; 12M = 6.2 ± 3.4 | NA | ELISA |
| Ali et al. 2020 | USA | RCT | n = 15; Age = 53 ± 2 years; 5 F and 10 M | Canagliflozin 100 mg | 16 weeks | B = NA; 16W = NA;  Δ = ND | B = 16 ± 23.24; 16W = 19.5 ± 23.24 | NA | RIA |
|  |  |  | n = 15; Age = 53 ± 3 years; 7 F and 8 M | Liraglutide 1.2 mg |  | B = NA; 16W = NA;  Δ = ND | B = 21.8 ± 30.98; 16W = 19.8 ± 30.98 |  |  |
|  |  |  | n = 15; Age = 51 ± 3 years; 9 F and 6 M | Canagliflozin 100 mg + liraglutide 1.2 mg |  | B = NA; 16W = NA;  Δ = ND | B = 20.6 ± 27.11; 16W = 22.1 ± 27.11 |  |  |
| Braga et al. 2021 | Brazil | PCS-S | n = 15; Age = 40.13 ± 10.8 years; 12 F and 3 M | RYGB | 3 months | B = 110.4 ± 16.7; 3M = 91.0 ± 14.6; Δ = -17.57% | B = 18.29 ± 13.27; 3M = 17.14 ± 16.18 | NA | Human Metabolic Hormone Magnetic Bead Panel, Milliplex MAP kit (Merck Millipore) |
| Camastra et al. 2013 | Italy | PCS-C | n = 12 | RYGB | 12 months | B = 139 ± 31.18; 12M = 91 ± 20.78; Δ = -34.53% | B = 19.8 ± 48.74; 12M = 9.19 ± 15.9 | NA | RIA |
|  |  |  | n = 15 | RYGB |  | B = 146 ± 23.24; 12M = 99 ± 19.37; Δ = -32.19% | B = 19.8 ± 44.45; 12M = 14.35 ± 25.56 |  |  |
| Capstick et al. 1997* |  | PCS-S | n = 14 | VLCD (Modifast) | 12 weeks | B = 108.9 ± 22.22; 12W = 94.5 ± 24.44; Δ = -13.22% | NA | Δ = -26.95 | High-Performance Liquid Chromatography (HPLC) |
| Casajoana et al. 2021 | Spain | RCT | n = 15; Age = 51.0 ± 7.7 years; 7 F and 8 M | mRYGB | 1 year | B = 103.01 ± 10.8; 5Y = 66.61 ± 10.7; Δ = -35.33% | B = 55.4 ± 75.6; 5Y = 26.6 ± 14.8 | NA | RIA |
|  |  |  | n = 15; Age = 49.2 ± 9.1 years; 5 F and 10 M | SG |  | B = 102.3 ± 10.7; 5Y = 74.95 ± 10.09; Δ = -26.73% | B = 48.2 ± 41.5; 5Y = 24.8 ± 35.9 |  |  |
|  |  |  | n = 15; Age = 49.7 ± 8.1 years; 3 F and 12 M | GCP |  | B = 105.53 ± 11.8; 5Y = 83.59 ± 13.56; Δ = -20.79% | B = 41.9 ± 53.3; 5Y = 35 ± 30.4 |  |  |
| Cersosimo et al. 2024 | USA | RCT | n = 25; Age = 49 ± 2 years; 16 F and 9 M | Dapagliflozin 10 mg + Exenatide 5 µg | 4 months | B = 92.1 ± 2.5; 4M = 84.5 ± 2.3; Δ = -8.25% | B = 12.05 ± 20; 4M = 11.77 ± 20 | NA | RIA |
| Clements et al. 2004 | USA | PCS-S | n = 20; Age = 40.3 ±7.9 years; 15 F and 5 M | RYGB | 12 weeks | B = 146.3 ± 34; 12W = 117.5 ± 26.9; Δ = -19.68% | B = 0.253 ± 0.0623; 12W = 0.241 ± 0.084 | NA | Immunochemi-luminometric assay |
| Coiro et al. 1988 | Italy | PCS-S | n = 9; Age = 30.2 ± 1.4 years; M | LCD (800-1000 kcal/day) | 6 - 8 months | B = 114.2 ± 21.30; 8M = 74.5 ± 11.40; Δ = -34.76% | B = 45.02 ± 11.02; 8M = 26.74 ± 5.77 | NA | RIA |
| deJonge et al. 2013 | Nether-lands, UK | PCS-S | n = 17; Age = 51 ± 2 years; 14 M and 3 F | DJBL | 24 weeks | B = 116.0 ± 5.8; 24W = NA; Δ = -10.95% | B = 30.38 ± 4.27; 24W = 22.6 ± 4.27 | NA | human diabetes bio-plex pro assay (Bio-Rad Laboratories BV) |
| Derosa et al. 2013 | Italy | RCT | n = 86; Age = 57.3 ± 7.7 years; 43 F and 43 M | Exenatide | 12 months | B = 89.8 ± 9.7; 12M = 82.6 ± 5.2; Δ = -8.02% | B = 63.9 ± 10.1; 12M = 33.2 ± 4.8 | NA | RIA |
|  |  |  | n = 85; Age = 56.7 ±7.3 years; 44 F and 41 M | Placebo |  | B = 90.5 ± 10.3; 12M = 88.2 ± 8.9; Δ = -2.54% | B = 62.5 ± 8.9; 12M = 47.9 ± 5.9 |  |  |
| Dirksen et al. 2013 | Denmark | PCS-S | n = 11; Age = 40.9 ± 10.8 years; 6 F and 5 M | RYGB | 3 months | B = 123.3 ± 6.2; 3M = 104.9 ± 5.5; Δ = -14.92% | B = 18.5 ± 3; 3M = 14.8 ± 2.2 | NA | RIA |
| Engelbrechtsen et al. 2016* | Denmark | RCT | n = 27; Age = 46 ± 2 years; 22 F and 5 M | VLCD (estimated daily energy needs subtracted by 600 kcal) + liraglutide 1.2 mg | 8 weeks | B = 98.6 ± 13.5; 8W = NA; Δ = ND | NA | Δ = -15% | nuclear magnetic resonance (NMR) spectroscopy |
| Fatima et al. 2022 | Norway | RCT | n = 53; Age = 48 ± 10 years | RYGB | 12 months | B = 124 ± 8.15; 12M = 89 ± 8.15; Δ = -28.23% | B = 23.9 ± 3.78; 12M = 12.6 ± 3.93 | NA | RIA |
|  |  |  | n = 53 | SG |  | B = 125 ± 8.15; 12M = 97 ± 8.15; Δ = -22.4% | B = 20.4 ± 3.78; 12M = 12.1 ± 3.78 |  |  |
| Fontes-Villalba et al. 2016 | Sweden | RCT | n = 6; Age = 63 ± 6 years; 2 F and 4 M | Paleolithic diet | 6 months | B = 92 ± 20; 6M = 84 ± 15; Δ = -8.7% | B = 124.8 ± 52.4; 6M = 123.6 ± 51 | NA | Bio-Plex pro human diabetes assay (Luminex-based magnetic bead assay) |
| Gougeon 2001 | Canada | PCS-C | n = 9; Age = 48 ± 1years; 5 F and 4 M, T2DM | VLCD (based on a casein-soy protein formula by Bariatrix: <460 kcal/day, 1.5g/kg protein, 10-15g carbs, 2g/day fat) | 28 days | B = 108 ± 30.0; 28D = NA; Δ = -9.17% | B = 24 ± 2; 28D = 22 ± 2 | NA | RIA |
|  |  |  | n = 7; Age = 42 ± 5 years; 6 F and 1 M | VLCD |  | B = 110 ± 26.46; 28D = NA; Δ = -8.64% | B = 22 ± 2; 28D = 20 ± 3 |  |  |
| Guldstrand et al. 2003 (1) | Sweden | PCS-S | n = 13; Age = 33.5 ± 4.2 years; F | Modified Mason vertical banded gastroplasty or jejuno-ileal bypass | at 25% WL (1 year) | B = 127.9 ± 20.1; 1Y = 94.4 ± 18.7; Δ = −25.81% | B = 13.68 ± 2.21; 1Y = 13.46 ± 2.73 | NA | RIA |
| Guldstrand et al. 2003 (2) | Sweden | PCS-S | n = 8; Age = 40 years; 7 F and 1 M | Vertical banded gastroplasty | 12.8 ± 5.6 months | B = 124.9 ± 12.2; 12M = 85.1 ± 6.0; Δ = -31.68% | B = 19.23 ± 2.7; 12M = 15.18 ± 3.07 | NA | RIA |
| Haas et al. 2022 | Sweden | PCS-S | n = 10; Age = 53 ± 8.9 years; 4 F and 6 M | VLCD (Modifast, <800 kcal/d) | 7 weeks | B = 125 ± 17.48; 7W = 110 ± 14.68; Δ = -12% | B = 47 ± 10.49; 7W = 34 ± 9.08 | NA | RIA |
| Heise et al. 2022 | Germany | RCT | n = 45; Age = 61.1 ± 7.1 years; 14 F and 31 M | Tirzepatide 15mg | 28 weeks | B = 94.1 ± 14.09; 28W = 83.4 ± 6.04; Δ = -11.9% | B = 11.8 ± 6.04; 28W = 9.1 ± 4.03 | NA | chemiluminescent sandwich immunoassay |
|  |  |  | n = 44; Age = 63.7 ± 5.9 years; 10 F and 34 M | Semaglutide 1mg |  | B = 92.2 ± 14.59; 28W = 87.7 ± 5.97; Δ = -7.38% | B = 12.3 ± 5.97; 28W = 9.9 ± 3.98 |  |  |
|  |  |  | n = 28; Age = 60.4 ± 7.6 years; 7 F and 21 M | Placebo (Control) |  | B = 99.1 ± 14.29; 28W = 94.6 ± 5.82; Δ = -4.54% | B = 15.2 ± 5.82; 28W = 13.6 ± 4.23 |  |  |
| Hoffer et al. 1984* | USA | RCT | n = 9; Age = 25 ± 4 years | Diet (1.5 g protein/kg ideal body weight) | 5 weeks | B = 84.0 ± 10; 5W = 79.0 ± 13.5; Δ = -5.95% | NA | Δ =  -43.59% | continuous primed tracer isotope infusion |
| Iepsen et al. 2016 | Denmark | PCS-S | n = 20; Age = 43.4 ± 9.6 years | VLCD (Cambridge diet, 810 kcal/d) | 8 weeks | B = 95.2 ± 8.1; 8W = 85 ± 10.6; Δ = -10.71% | B = 1.21 ± 0.14; 8W = 1.29 ± 0.11 | NA | RIA |
| Jacobsen et al. 2013 | Denmark | PCS-S | n = 10; Age = NA | RYGB | 3 months | B = NA; 3M = NA; Δ = ND | B = 6.5 ± 3.79; 3M = 5.1 ± 5.69 | NA | RIA |
| Jørgensen et al. 2012 | Denmark | PCS-C | n = 13; Age = 52 ± 9 years; 6 F and 7 M | RYGB | 1 year | B = 128.8 ± 18.39; 12M = 100.8 ± 70.32; Δ = -21.75% | B = 13 ± 7; 12M = 8 ± 4 | NA | RIA |
|  |  |  | n = 12; Age = 43 ± 13 years; 9 F and 3 M; T2DM | RYGB |  | B = 126.6 ± 53.38; 12M = 94.7 ± 55.42; Δ = -25.22% | B = 10 ± 3.0; 12M = 2 ± 2.0 |  |  |
| Jorsal et al. 2019 | Denmark | PCS-S | n = 8; Age = 48 ± 5.6 years; 6 F and 2 M | RYGB | 3 months | B = 128 ± 21.8; 3M = NA; Δ = -25.77% | B = 5.13 ± 1.05; 3M = 5.57 ± 0.97 | NA | RIA and ELISA |
| Jensen et al. 2023 | Denmark | RCT | n = 49; Age = NA | Liraglutide 3.0 mg/day + exercise | 8 weeks | B = NA; 8W = NA; Δ = ND | B = 7.9; 8W = 7 ± 5.3 | NA | RIA |
|  |  |  | n = 49; Age = NA | Placebo (Control) |  | B = NA; 8W = NA; Δ = ND | B = 7.6 ± 3.2; 8W = 8.1 ± 5.5 |  |  |
| Kahn et al. 2021 | USA | RCT | n = 68; Age = 54.0 ± 8.1 years; 29 F and 39 M | Liraglutide + MET | 12 months | B = 104.2 ± 21; 12M = 97.4 ± 20.7; Δ = -6.52% | B = 8.82 ± 5.11; 12M = 7.12 ± 4.8 | NA | ELISA |
|  |  |  | n = 62; Age = 52.9 ± 9.9 years; 24 F and 38 M | Placebo (Control) |  | B = 101.3 ± 19.6; 12M = 99.2 ± 19.6; Δ = -2.07% | B = 7.68 ± 3.82; 12M = 8.58 ± 5.12 |  |  |
| Kalinowski et al. 2017 | Poland | RCT | n = 36; Age = 44.9 ± 10.6 years; 26 F and 10 M | SG | 12 months | B = 127.9 ± 17.6; 12M = 90.6 ± 15.9; Δ = -29.16% | B = 72 ± 38; 12M = 57.4 ± 31 | NA | ELISA |
|  |  |  | n = 36; Age = 43.9 ± 10.8 years; 23 F and 13 M | RYGB |  | B = 141.2 ± 20.6; 12M = 98.2 ± 18.1; Δ = -30.45% | B = 65 ± 26.9; 12M = 60 ± 30 |  |  |
| Kashyap et al. 2013 | USA | RCT | n = 17; Age = 50 ± 8.4 years; 8 F and 9 M | Intensive medical therapy (control) | 24 months | B = 107.9 ± 14.5; 24M = 107.4 ± 14.9; Δ = -0.46% | B = 25.5 ± 26.67; 24M = 18.1 ± 18.52 | NA | RIA |
|  |  |  | n = 18; Age = 47.9 ± 9.7 years; 8 F and 10 M | RYGB |  | B = 105.3 ± 13.6; 24M = 79.9 ± 11.7; Δ = -24.12% | B = 28.3 ± 42.22; 24M = 18.5 ± 20 |  |  |
|  |  |  | n = 19; Age = 47.5 ± 10.0 years; 16 F and 3 M | SG |  | B = 100 ± 16.5; 24M = 77.5 ± 14.3; Δ = -22.5% | B = 23.8 ± 29.63; 24M = 16.4 ± 11.85 |  |  |
| Katsogiannos et al. 2020 | Sweden | RCT | n = 13; Age = 55 ± 9 years; 10 F and 3 M | RYGB | 24 weeks | B = 100 ± 16.5; 24W = 77 ± 10; Δ = -23% | B = 8 ± 8; 24W = 10 ± 13 | NA | ELISA |
|  |  |  | n = 6; Age = 49 ± 5 years; 2 F and 4 M | Standard-of-care (Control) |  | B = 116 ± 8; 24W = 115 ± 9; Δ = -1.72% | B = 7 ± 5; 24W = 8 ± 6 |  |  |
| Kjeldsen et al. 2023 (1)* | Denmark | RCT | n = 14; Age = 63 ± 8 years; 36 F and 64 M | Isocaloric CD diet (Control) |  | B = 88 ± 22; 6W = NA; Δ = -0.91 % | B = 10.5 ± 12; 6W = 10.5 |  | ELISA and L-Amino Acid Assay kit (Abcam, ab65347) |
|  |  |  | n = 14; Age = 65 ± 6 years; 21 F and 79 M | Isocaloric CRHP diet |  | B = 90 ± 15; 6W = NA; Δ = -1.56 % | B = 8.7 ± 5; 6W = 8 | Δ =  -10.03% |  |
| Kjeldsen et al. 2023 (2)*^/^** | Denmark | RCT | n = 32; Age = 67 ± 9 years; 53 F and 47 M | Hypocaloric CD diet (Control) | 6 weeks | B = 98 ± 25; 6W = NA; Δ = -5.92% | B = 8 ± 4.2; 6W = 7.2 |  | ELISA and L-Amino Acid Assay kit (Abcam, ab65347) |
|  |  |  | n = 34; Age = 66 ± 7 years; 41 F and 59 M | Hypocaloric CRHP diet |  | B = 98 ± 14; 6W = NA; Δ = -5.92% | B = 8.5 ± 4.4; 6W = 6.4 | Δ =  -18.22% |  |
| Laferrere et al. 2008 | USA | PCS-C | n = 9; Age = 44.56 ± 9.65 years; F | RYGB | 1 months | B = 113.2 ± 15.5; 1M = 103.2 ± 16.6; Δ = -8.83% | B = 18.91 ± 4.3; 1M = 19.23 ± 6.26 | NA | RIA |
|  |  |  | n = 10; Age = 46.8 ± 7.24 years; F | LCD (1000 kcal/d) | after 10 kg WL (4 - 8 weeks) | B = 110.9 ± 10.2; 8W = 101.1 ± 9.5; Δ = -8.84% | B = 20.55 ± 3.04; 8W = 16.79 ± 4.59 |  |  |
| Lopes et al. 2016* | Brazil | PCS-S | n = 10 | RYGB | 12 months | BMI at baseline = 32.38 ± 2.11; 12M = 25.48 ± 1.85 | NA | Δ = -18% | time-resolved ¹H Nuclear Magnetic Resonance (NMR) |
| Meryn et al. 1986 | USA | PCS-S | n = 6; Age = NA; 3 F and 3 M | RYGB | 6 - 9 months | B = 163 ± 66; 6-9M = 121 ± 49; Δ = -25.77% | B = 28 ± 5.5; 6-9M = 20 ± 2.6 | NA | RIA |
| Nannipieri et al. 2013 | Italy | PCS-C | n = 23; Age = 51 ± 9 years | RYGB | 12 months | B = 113 ± 20; 12M = 90 ± 13; Δ = -30.97% | B = 17.22 ± 6.31; 12M = 14.92 ± 13.2 | NA | RIA |
|  |  |  | n = 12; Age = 57 ± 7 years | SG |  | B = 124 ± 18; 12M = 95 ± 11; Δ = -8.84% | B = 13.77 ± 10.04; 12M = 16.64 ± 10.62 |  |  |
| Nguyen et al. 2015 | USA | RCT | n = 34; Age = 50 ± 1 years; 21 F and 13 M | RYGB | 12 months | B = 103.2 ± 13.41; 12M = 74.2 ± 12.83; Δ = -28.09% | B = 24 ± 28.57; 12M = 19.1 ± 19.24 | NA | RIA |
|  |  |  | n = 34; Age = 50 ± 1 years; 14 F and 20 M | lifestyle modification + intensive medical management |  | B = 105.7 ± 16.91; 12M = 96.1 ± 17.49; Δ = -9.08% | B = 26.7 ± 43.73; 12M = 23.2 ± 29.74 |  |  |
| Otten et al. 2019 | Sweden | RCT | n = 26; Age = 61 ± 1 years | Paleolithic diet (high protein, low carb) | 24 months | B = 85.5 ± 10.71; 24M = 77.3 ± 11.73; Δ = -9.59% | B = 5 ± 1.53; 24M = 5.7 ± 2.04 | NA | RIA |
|  |  |  | n = 23; Age = 61 ± 1 years | Control (nordic diet) |  | B = 84.9 ± 7.68; 24M = 80.1 ± 9.6; Δ = -5.65% | B = 5.5 ± 1.92; 24M = 6.8 ± 1.92 |  |  |
| Otten et al. 2021*^/^** | USA | RCT | n = 15; Age = 60 ± 8.15 years; 5 F and 10 M | Paleolithic diet | 12 weeks | B = 90.0 ± 13.78; 12W = NA; Δ = -7.89% | B = 13 ± 5.19; 12W = NA; Δ = -3 ± 2.96 |  | RIA and LC-electrospray ionization-MS/MS |
|  |  |  | n = 14; Age = 61 ± 5.93 years; 5 F and 9 M | Paleolithic diet + exercise |  | B = 97.3 ± 22.59; 12W = NA; Δ = -7.3% | B = 11 ± 3.7; 12W = NA; Δ = -1 ± 5.19 | Δ = 1.36% |  |
| Pedersen et al. 2020*^/^** | Denmark | PCS-C | n = 17; Age = 44 ± 11.85 years; 12 F and 5 M; OBE no steatosis | RYGB or SG | 12 months | B = 121 ± 17.04; 12M = 90 ± 12.59; Δ = -25.62% | B = 8 ± 4.07; 12M = 6 ± 1.85 | Δ =  -17.32% | ELISA |
|  |  |  | n = 8; Age = 42 ± 9.63 years; 5 F and 3 M; OBE + steatosis | RYGB or SG |  | B = 136 ± 16.3; 12M = 96 ± 15.56; Δ = -29.41% | B = 7.5 ± 2.81; 12M = 4.5 ± 2.96 |  |  |
|  |  |  | n = 8; Age = 44 ± 9.63 years; 2 F and 6 M; OBE-NASH-Fib | RYGB or SG |  | B = 115 ± 20.0; 12M = 90 ± 14.81; Δ = -21.74% | B = 7.5 ± 2.81; 12M = 7 ± 3.56 |  |  |
| Pirro et al. 2021*^/^** | USA | RCT | n = 51; Age = 56.5 ± 9.1 years; 22 F and 22 M | Placebo (Control) | 26 weeks | B = 91.5 ± 23.1; 26W = NA; Δ = -0.44% | B = 13.7 ± 7.0; 26W = NA; Δ = +10.22% |  | ELISA and LC-MS with relative quantification |
|  |  |  | n = 55; Age = 57.8 ± 8.5 years; 19 F and 29 M | LY3298176 5 mg once weekly |  | B = 92.8 ± 19.0; 26W = NA; Δ = -5.17% | B = 11.9 ± 7.19; 26W = NA; Δ = −14.29% | Δ = -11.5% |  |
|  |  |  | n = 52; Age = 56.1 ± 10.5 years; 18 F and 23 M | LY3298176 10 mg once weekly |  | B = 92.7 ± 19.5; 26W = NA; Δ = -9.39% | B = 12.7 ± 7.21; 26W = NA; Δ = −14.17% | Δ = -13.2% |  |
|  |  |  | n = 53; Age = 55.0 ± 7.8 years; 24 F and 21 M | LY3298176 15 mg once weekly |  | B = 89.1 ± 22.7; 26W = NA; Δ = -12.68% | B = 12.1 ± 7.72; 26W = NA; Δ = −28.10% | Δ = -14.4% |  |
| Rohde et al. 2017 | Denmark | PCS-C | n = 10; Age = 49 ± 24.44 years | DJBL | 26 weeks | B = 101.1 ± 20.89; 26W = NA; Δ = -6.73% | B = 6 ± 2.22; 26W = 5.0 ± 2.96 | NA | RIA |
|  |  |  | n = 9; Age = 50 ± 20.0 years; T2DM |  |  | B = 103.7 ± 37.78; 26W = NA; Δ = -5.98% | B = 9 ± 2.22; 26W = 6.0 ± 2.96 |  |  |
| Sathananthan et al. 2015 | USA | PCS-S | n = 9; Age = 58 ± 3 years; 7 F and 2 M | Caloric restriction | 12 weeks | B = 114 ± 6; 12W = 101 ± 6; Δ = -12.98% | B = 26.11 ± 3.44; 12W = 20.38 ± 2.58 | NA | RIA |
| Steven et al. 2016 | UK | PCS-C | n = 16; Age = 49.1 ± 1.6 years; 11 F and 7 M; T2DM | RYGB | 8 weeks | B = 121.1 ± 23.9; X = 104.5 ± 2.7; Δ =-13.71 % | B = 21.41 ± 11.25; 8W = 16.64 ± 9.53 | NA | ELISA |
|  |  |  | n = 9; Age = 46.3 ± 2.1 years; 7 F and 2 M |  |  | B = 114.5 ± 5.0; X = 99.7 ± 4.6; Δ =-12.91 % | B = 14.18 ± 4.3; 8W = 13.8 ± 4.3 |  |  |
| Swarbrick et al. 2008 | USA | PCS-S | n = 19; Age = 41 ± 2 years; F | RYGB | 12 months | B = 126.4 ± 3.6; 12M = 85.9 ± 2.8; Δ = -32.04% | B = 28.13 ± 13.76; 12M = 18.37 ± 6.26 | NA | RIA |
| Tabasi et al. 2021 | Iran | PCS-S | n = 126; Age = NA; 96 F and 30 M | LSG | 12 months | B = 121.4 ± 23.9; 12M = 82.51 ± 15.77; Δ = -32.03% | B = 31.51 ± 5.48; 12M = 21.17 ± 3 | NA | ELISA |
| Umeda et al. 2011 | Brazil | PCS-S | n = 10 | RYGB | 90 days | B = 103.3 ± 16.4; 90D = 86.0 ± 12.0; Δ = -16.75% | B = 22.39 ± 5.89; 90D = 24.88 ± 5.85 | NA | ELISA |
| Vetter et al. 2015 | USA | PCS-C | n = 10; Age = 54.0 ± 6.6 years; 9 F and 1 M | RYGB | 58.9 + 12.1 days (until 10% WL) | B = 120.5 ± 23.9; 59D = 108.5 ± 6.3; Δ = -9.96% | B = 17.65 ± 1.81; 59D = 12.2 ± 1.26 | NA | RIA |
|  |  |  | n = 10; Age = 51.8 ± 11.6 years; 5 F and 5 M | Intensive lifestyle modification | 85.5 + 24.4 days (until 10% WL) | B = 122.0 ± 20.6; 85D = 109.4 ± 6.3; Δ = -10.33% | B = 18.28 ± 2.07; 85D = 13.46 ± 1.26 |  |  |
| Vilarrasa et al. 2017 | Spain | PCS-S | n = 21; Age = 54.1 ± 9.5 years; 9 F and 12 M | DJBL | 12 months | B = 91.2 ± 12.2; 12M = NA; Δ = -14.91% | B = 30.79 ± 26.2; 12M = 42.73 ± 27.96 | NA | enzyme immunoassay (Yanahaira) |
| Villareal et al. 2008 | USA | RCT | n = 15; Age = 69 ± 1 years; 11 F and 4 M | Calorie restriction + exercise vs control | 26 weeks | B = 99 ± 15.49; 26W = NA; Δ = -9.09% | B = 43.05 ± 11.11; 26W = 40.17 ± 5.5 | NA | RIA |
| Yazawa et al. 2023*^/^** | Japan | RCT | n = 9; Age = 36.2 ± 8.5 years; M | Placebo (Control) | 16 weeks | B = 76.6 ± 7.1; 16W = NA; Δ = -1.02% | B = 51.6 ± 9.7; 16W = NA; Δ = −1.71% |  | ELISA |
|  |  |  | n = 9; Age = 31.9 ± 5.8 years; M | BI 456906 (GLP-1RA) 4.8 mg once weekly |  | B = 72.2 ± 5.3; 16W = NA; Δ = -11.16% | B = 50.3 ± 4.4; 16W = NA; Δ = −23.06% | Δ = -27.2% |  |
|  |  |  | n = 9; Age = 31.6 ± 7.5 years; M | BI 456906 (GLP-1RA) 2.4 mg twice weekly |  | B = 77.8 ± 7.9; 16W = NA; Δ = -8.34% | B = 49.3 ± 7.53; 16W = NA; Δ = −22.11% | Δ = -45.7% |  |

**Table S1.** Summary of studies where glucagon and/or alanine was measured alongside body weight. Concentrations of glucagon and alanine have been converted to be presented in pmol/L where necessary. The change of alanine is shown as percentage (%). All results are presented as mean ± SD. *B*, Baseline; *Δ*, Change from baseline to endpoint (%); *PCS-S*, Single-arm prospective cohort study; *PCS-C*, controlled prospective cohort study; *(m)RYGB*, modified Roux-en-Y gastric bypass; *(L)SG*, (Laparoscopic) Sleeve gastrectomy; *GCP*, Gastric Curvature Plication; *DJBL*, Duodenal-Jejunal Bypass Liner; CD, conventional diabetes diet; *CRHP*, Carbohydrate-reduced high-protein diet; *(V)LCD*, (Very) Low-calorie diet***; *MET*, Metformin; *ELISA*, Enzyme-linked immunosorbent assay; *RIA*, Radioimmunoassay; *LC–MS(/MS)*, Liquid chromatography(–tandem) mass spectrometry

*Studies reporting on alanine

**Studies reporting on both alanine and glucagon

***LCD defined as 800-1200 kcal/day; VLCD defined as <800 kcal/day.

**Table S2. Risk of bias assessment of included non-randomised studies using MINORS**

**Table S2.** Risk of bias assessment of included non-randomised studies using MINORS.

**Figure S1.** Risk of bias assessment of included RCTs using the Cochrane RoB 2 tool and proportion of studies judged as low risk, some concerns, or high risk of bias across each domain and for overall risk of bias. Visualised with robvis tool.^1^

**A	B**

**Figure S2. (A)** Forest plot presenting meta-analysis of effect sizes (ES) with 95% confidence intervals (CI) for changes in absolute body weight (kg) across all intervention arms (n = 69). The pooled overall effect estimate was derived using DerSirmonian-Laird (DL) method. Heterogeneity was measured by I^2^. Weight corresponds to the percentage of contribution of each intervention to the overall effect. **(B)** Forest plot presenting random-effects meta-analysis of RCTs, using the restricted maximum likelihood (REML) method. The plot presents the mean difference and standard deviation (SD) in absolute body weight (kg) between treatment and control groups, with 95% confidence intervals (CI). Each square represents an individual comparison, with the size proportional to its weight in the analysis, and horizontal lines denote 95% CI. The diamond indicates the pooled overall effect (mean difference –10.03 kg; 95% CI [–13.48, –6.57]; *p* < 0.001). Between-study heterogeneity was substantial (τ² = 44.29, I² = 95.1%, *p* < 0.001).

**A	B**

**Figure S3. (A)** Forest plot presenting meta-analysis of effect sizes (ES) with 95% confidence intervals (CI) for changes in fasting plasma glucagon (pmol/L) across all intervention arms (n = 69). The pooled overall effect estimate was derived using DerSirmonian-Laird (DL) method. Heterogeneity was measured by I2. Weight corresponds to the percentage of contribution of each intervention to the overall effect. **(B)** Forest plot presenting random-effects meta-analysis of RCTs, using the restricted maximum likelihood (REML) approach. The plot presents the mean difference and standard deviation (SD) in fasting plasma glucagon (pmol/L) between treatment and control groups, with 95% confidence intervals (CI). Each square represents an individual comparison, with the size proportional to its weight (%) in the analysis, and horizontal lines denote 95% CI. The diamond indicates the pooled overall effect (mean difference –4.45 pmol/L; 95% CI [–6.83, –2.07]; p < 0.001). Between-study heterogeneity was substantial (τ² = 44.29, I² = 95.1%, p < 0.001).

 **A	B**

**Figure S4.** Forest plots presenting meta-analyses of the mean difference in (A) absolute body weight (kg) and (B) fasting alanine levels (% change from baseline) following weight loss interventions. Dietary interventions are shown in green, pharmacological interventions in blue and surgical interventions in red. Heterogeneity was measured by I^2^. Weight corresponds to the percentage of contribution of each intervention to the overall effect.

**References**

1. McGuinness LA, Higgins JPT. Risk-of-bias VISualization (robvis): An R package and Shiny web app for visualizing risk-of-bias assessments.

*Research Synthesis Methods*. 2020/04/26 2020;n/a(n/a)doi:10.1002/jrsm.1411
